# The circulation of methicillin-resistant *Staphylococcus aureus* between humans, horses and the environment at the equine clinic

**DOI:** 10.1093/jac/dkae303

**Published:** 2024-08-30

**Authors:** Aneta Papouskova, Zuzana Drabkova, Marie Brajerova, Marcela Krutova, Alois Cizek, Jan Tkadlec

**Affiliations:** Institute of Infectious Diseases and Microbiology, Faculty of Veterinary Medicine, University of Veterinary Sciences Brno, Brno, Czech Republic; Equine Clinic, Faculty of Veterinary Medicine, University of Veterinary Sciences Brno, Brno, Czech Republic; Department of Medical Microbiology, 2nd Faculty of Medicine, Charles University and Motol University Hospital, Prague, Czech Republic; Department of Medical Microbiology, 2nd Faculty of Medicine, Charles University and Motol University Hospital, Prague, Czech Republic; Institute of Infectious Diseases and Microbiology, Faculty of Veterinary Medicine, University of Veterinary Sciences Brno, Brno, Czech Republic; Department of Medical Microbiology, 2nd Faculty of Medicine, Charles University and Motol University Hospital, Prague, Czech Republic

## Abstract

**Objectives:**

We performed a retrospective analysis of MRSA isolates collected at the university equine clinic including clinical isolates from 2008 to 2021 and screening environmental, equine and personnel isolates from 2016.

**Methods:**

Screening and clinical samples were cultured on Brilliance MRSA 2 and Columbia agar (Oxoid), respectively, with enrichment for environmental samples. Antimicrobial susceptibility was assessed by disc diffusion. All the isolates were characterized by *spa* typing. Eighteen selected isolates were subjected to WGS with subsequent wgMLST clonal analysis.

**Results:**

Among 75 MRSA isolates, five *spa* types were identified, the majority (*n* = 67; 89.33%) was t011. All isolates were resistant to cefoxitin and ampicillin and carried the *mecA* gene. In addition, the isolates were resistant to tetracycline (*n* = 74; 98.67%), gentamicin (*n* = 70; 93.33%), enrofloxacin (*n* = 54; 72.00%), sulfamethoxazole-trimethoprim (*n* = 5; 6.67%) and lincomycin (*n* = 3; 4.00%) with corresponding genetic markers for the resistance detected in the sequenced isolates. All 18 sequenced isolates belonged to ST398, 16 carried SCC*mec* type IVa and two carried SCC*mec* type Vc (5C2&5). Further, isolates carried *aur*, *hlgA*, *hlgB* and *hlgC* virulence genes, and five isolates carried *sak* and *scn* genes, which are part of the immune evasion cluster. Close genetic relatedness was found between isolates from the staff of the clinic and clinical samples of horses.

**Conclusions:**

Repeated introduction and long-term persistence of the equine LA-MRSA subclone (ST398-MRSA-IVa/Vc(5C2&5), t011) among the infected horses at the equine clinic with the colonization of personnel, and the environment contamination that might contribute to transmission were observed.

## Introduction

In both human and veterinary medicine, MRSA represents a serious clinical problem with a limited range of available therapeutic options. Among the so-called livestock-associated MRSA (LA-MRSA), the most common clonal lineage in Europe is ST398. European ST398 consists of several sublineages characterized by t011 or t034 *spa* types and the presence of SCC*mec* types IV or V.^1^ LA-MRSA ST398 is characterized by the presence of the Tn*916* transposon carrying *tet*(M), which provides resistance to tetracycline, and by the loss of a temperate ϕSa3 prophage carrying human-specific virulence factors, namely, the *sak* and *scn* genes, which are part of the immune evasion cluster (IEC).^2^ This lineage, which was originally found in swine, has spread to poultry, cattle, horses and other livestock and pet animals.^2,3^ Importantly, LA-MRSA ST398 retains the capacity to colonize or infect humans.^4,5^

This study aimed to analyse the clonal structure of clinical MRSA isolates (2008–21) at the university equine clinic and to compare selected equine MRSA with isolates from the environment and personnel obtained during a single sampling in 2016 to investigate the transmission routes of MRSA.

## Materials and methods

### Ethics

Staff sampling was performed under internal regulations of the Faculty of Veterinary Medicine, University of Veterinary Sciences Brno. All human subjects provided written informed consent.

### MRSA isolates

All clinical MRSA isolates from horses hospitalized at the equine clinic of the University of Veterinary Sciences (VETUNI, Brno, Czech Republic) were collected after culture of samples on Columbia agar with 5% sheep blood (Oxoid, Basingstoke, UK) at 37°C for 24 h.

Environmental samples^6^ were enriched in brain–heart infusion broth supplemented with 6.5% NaCl (Oxoid) for 24 h at 37°C and subsequently streaked onto Brilliance MRSA 2 agar (Oxoid). The nasal swabs of the horses and palm swabs of staff were streaked directly onto Brilliance MRSA 2 agar. The agar plates were incubated for 24 h at 37°C. Species identification was performed by MALDI-TOF/MS (Bruker Daltonik, Bremen, Germany).

### Antimicrobial susceptibility testing, MRSA confirmation and spa typing

The antimicrobial susceptibility was determined by a disk diffusion test according to CLSI.^7,8^ The *mecA* gene and gene for Panton-Valentine leukocidin (PVL) were detected by PCR.^9^*spa* typing was performed as described previously.^10^

### Whole-genome sequencing

DNA from a single colony was isolated using the MasterPure Complete DNA and RNA Purification Kit (Biosearch Technologies, Hoddesdon, UK). The DNA sequencing library was prepared with the Nextera XT DNA Library Preparation Kit (Illumina, San Diego, CA, USA). The pooled libraries were sequenced on an Illumina HiSeq X Ten sequencer (Illumina). Raw reads were assembled using SPAdes v 3.15.5.^11^

Long-read sequencing using MinION (Oxford Nanopore Technologies, Oxford, UK) was performed as described previously.^12^ The hybrid assembly of long and short reads was performed using Flye assembler v2.9.2,^13^ Medaca v1.9.1 for long-read polishing (Oxford Nanopore Technologies) and Polypolish v0.5.0 for short-read polishing.^14^

### Bioinformatics

The presence of resistance genes, plasmids, virulence genes, SCC*mec* typing and MLST were investigated using tools available at the Center for Genomic Epidemiology website (https://www.genomicepidemiology.org/).

The genetic relatedness was determined using whole-genome MLST (wgMLST) and the minimum spanning tree was constructed using Bionumerics v8.1 (bioMérieux, Applied Maths, Sint-Martens-Latem, Belgium) using 3897 loci. A threshold of ≤24 allelic differences was used to assume close genetic relatedness.^15^

Raw reads were submitted to the NCBI Sequence Read Archive under BioProject accession number PRJNA1057774. Additionally, hybrid genome assemblies of isolates LA7, LA23, LA31, LA52 and LA72 were submitted under accession numbers: JAZHCX000000000, JAZHCW000000000, CP144271, CP144269-CP144270 and JAZHCV000000000, respectively.

## Results

### Isolates

From February 2008 to September 2021 a total of 34 clinical MRSA (see Table S1 available as Supplementary data at *JAC* Online) were collected. In addition, 41 screening MRSA isolates were collected between 6^th^ September and 17^th^ October 2016 from the environment (*n* = 28), staff (*n* = 11; 11/21 tested employees) and horses (*n* = 2) (Table 1).

**Table 1.** MRSA isolates from the equine clinic 2008–21—origin, resistance and spa typing

Year	Origin (no.)	Resistance profiles^a^	*spa* types
2008–2010	Clinic (6)	CN;TET; SXT (4)	t064
		TET; MY (2)	t011
2011–2015	Clinic (8)	TET (1)	t011
		CN; TET (4)	t011
		CN; TET; ENR (3)	t011
2016–2021	Clinic (20)	CN; TET; ENR (19)	t011
		CN; TET (1)	t6867
2016	Environment (28)	CN, TET, ENR (19)	t011
		CN, ENR (1)	t011
		CN, TET (6)	t011 (5); t2346 (1)
		TET, SXT (1)	t064
		TET, MY, ENR (1)	t034
	Personnel (11)	CN, TET, ENR (9)	t011
		CN, TET (2)	t011
	Horse—colonization (2)	CN, TET, ENR (2)	t011

CN, gentamicin; TET, tetracycline; MY, lincomycin; ENR, enrofloxacin; SXT, sulfamethoxazole-trimethoprim.

^a^All isolates displayed resistance to cefoxitin (FOX) and ampicillin (AMP).

### Antimicrobial susceptibility testing

All isolates were resistant to cefoxitin and carried the *mecA* gene. In addition, the isolates were found to be resistant to tetracycline (*n* = 74; 98.67%), gentamicin (*n* = 70; 93.33%), enrofloxacin (*n* = 54; 72.00%), sulfamethoxazole-trimethoprim (*n* = 5; 6.67%) and lincomycin (*n* = 3; 4.00%) (Table S1).

### Clonal analysis

The majority of isolates (*n* = 67) were t011. Other *spa* types were less frequent (Table 1), and none of the isolates carried PVL. WGS was performed to determine the genetic relatedness of 18 selected t011 isolates. Eleven isolates were from infected horses collected from 2008 to 2021, and seven isolates were collected in 2016 from personnel (*n* = 3) and the environment (*n* = 4). Five of these isolates were sequenced by long-read sequencing to obtain information on resistance gene localization in detected plasmids.

All 18 sequenced isolates were ST398. Except for two isolates from 2008 and 2010 that carried SCC*mec* type Vc (5C2&5), all other isolates carried SCC*mec* type IVa (Table S2).

Further, mutations associated with resistance to enrofloxacin (GrlA S80F and GyrA S84L) and genes associated with resistance to tetracycline (*tet*(M); *tet*(K)), gentamicin (*aac*(6’)*-aph*(2'’); *aph*(2'’)*-Ia*), lincomycin (*vga*(A)_LC_) and trimethoprim (*dfrK*) were found (Table S2).

**Figure 1.**
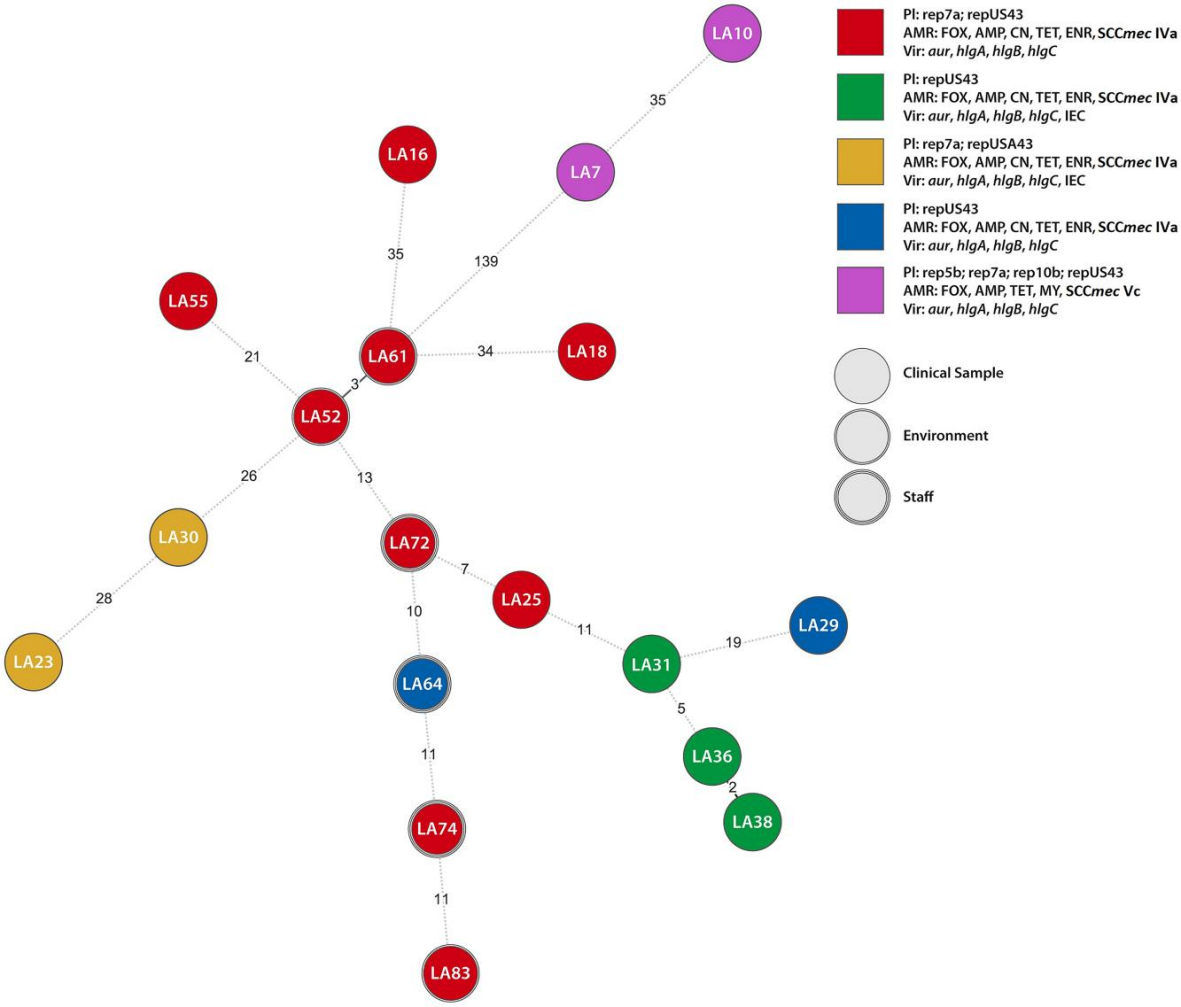
Minimum spanning tree (MST) based on wgMLST analysis for 18 equine MRSA. The minimum spanning tree was constructed using Bionumerics v8.1 (bioMérieux, Applied Maths) using 3897 loci. The nodes represent genomes of individual isolates and branch labels represent allelic distances between neighbouring isolates. The colour and the shape of the node are used to indicate different genetic content (plasmids, virulence and resistance genes) and isolate origin (clinical sample, environment, staff), respectively. Pl, plasmids; AMR, antimicrobial resistance; FOX, cefoxitin; AMP, ampicillin; CN, gentamicin; TET, tetracycline; MY, lincomycin; ENR, enrofloxacin; SXT, sulfamethoxazole-trimethoprim; Vir, virulence genes. This figure appears in colour in the online version of *JAC* and in black and white in the print version of *JAC*.

Regarding virulence factors, all sequenced isolates carried the genes *aur*, *hlgA*, *hlgB* and *hlgC*. In addition, five isolates carried *sak* and *scn* genes that are part of the IEC (Figure 1). Other virulence genes were not detected (see Table S2).

wgMLST analysis revealed allelic differences of 2–166 between the isolates (Figure 1, Table S3). The closest genetic relatedness between the animal (LA25) and human (LA72) isolates was seven allelic differences, and between the environmental and human isolates, there were 11 allele differences (LA83 and LA74).

For five isolates, the complete genome was obtained to investigate the plasmid content. The plasmids were small and contained only genes for plasmid survival. The only resistance genes carried by the plasmid were aminoglycoside 6-adenylyltransferase *str* on the rep7a plasmid in four of five isolates (in LA7 in two copies) and *vga*(A)_LC_ carried by the rep5a/rep10b plasmids in one isolate. In all the isolates, the repUS43 plasmid was found to be incorporated into the genome. The length and gene content of the plasmids are provided in Table S4.

## Discussion

The spread of MRSA among equine clinics represents a significant burden on the quality of equine healthcare but also, a possible source for the spread of MRSA to humans in contact with horses.^16^

The majority (*n* = 67; 89.3%) of isolates in our study belonged to *spa* type t011, a typical equine MRSA in central Europe, characterized by ST398, aminoglycoside resistance, and SCC*mec* type IV.^4,5,17^

The close genetic relatedness between isolates from infected horses, the environment and clinical personnel was confirmed by wgMLST analysis in selected isolates. In general, the allelic difference between neighbouring isolates in our collection rarely exceeded the suggested 24 allele threshold for a close genetic relatedness of *Staphylococcus aureus.*^15^ Two isolates from 2008 were separated from the other isolates by 139–166 allele differences and carried different SCC*mec* type Vc (5C2&5), which is a composite element containing class C2 *mec* gene complex and type 5 *ccr* gene complex with two copies of *ccrC*.

Considering that most of the infections in this study were related to surgical procedures, human-to-horse contact seems to be the possible route of MRSA transmission but close genetic relatedness was also observed between environmental and human isolates. A previous Czech study focusing on MRSA carriage among 134 veterinarians revealed that seven of nine MRSA carriers were colonized by t011-IV or a closely related (t2330-IV).^18^ Similarly, t011-IV was the most common among MRSA colonizing personnel of an equine clinic in Germany, 2012–14, but human infections were rare.^5^ Among the 1900 human MRSA isolates collected between 2004 and 2008 in two regions in Austria, a total of 41 (2.2%) were ST398 MRSA and 87.8% (*n* = 36) were t011.^19^ Only two t011-IVa (0.45%) were detected among 441 human MRSA isolates from Czech hospitals between 2017 and 2018.^20^

The loss of temperate ϕSa3 prophage carrying an IEC by CC398 is associated with animal host adaptation.^2^ In our study, five sequenced t011 isolates carried *sak* and *scn* genes that are part of the IEC. However, reacquisitions of IEC by CC398-MRSA were described.^2^

Frequent detection of resistance to tetracycline, aminoglycosides and quinolones corresponded to studies of equine MRSA isolates in Hungary and Germany.^4,5^ Enrofloxacin resistance was associated with the presence of parallel mutations in topoisomerase IV (GrlA S80F) and gyrase (GyrA S84L). Two isolates carried a single mutation in topoisomerase IV (GrlA S80F) and were susceptible to enrofloxacin. In addition, the corresponding aminoglycoside resistance genes were not identified in nine gentamicin-resistant isolates, and the mechanism of resistance remains unknown.

This study has several limitations. Environmental and human MRSA were collected at a single time point and the number of sequenced isolates was limited not allowing for direct confirmation of MRSA transmission between humans/environment and horses.

In conclusion, this study provides evidence of the long-term presence of equine ST398-MRSA clone (ST398-MRSA-IVa) with limited genetic diversity but also possible repeated introduction of the same clone (ST398-MRSA-V) among horses at the equine clinic. Environmental contamination and personnel colonization might contribute to MRSA transmission.

## Supplementary Material

dkae303_Supplementary_Data

## Supplementary data

Supplementary Tables S1–S4 are available as Supplementary data at *JAC* Online.
